# A review of 177Lu dosimetry workflows: how to reduce the imaging workloads?

**DOI:** 10.1186/s40658-024-00658-8

**Published:** 2024-07-18

**Authors:** Laure Vergnaud, Yuni K. Dewaraja, Anne-Laure Giraudet, Jean-Noël Badel, David Sarrut

**Affiliations:** 1 CREATIS; CNRS UMR 5220; INSERM U 1044, Université de Lyon; INSA-Lyon; Université Lyon 1, Lyon, France; 2 Department of Radiology, University of Michigan, Ann Arbor, USA; 3 Centre de lutte contre le cancer Léon Bérard, Lyon, France

**Keywords:** Review, Dosimetry, Single time-point, $^{177}{\hbox {Lu}}$

## Abstract

$^{177}{\hbox {Lu}}$ radiopharmaceutical therapy is a standardized systemic treatment, with a typical dose of 7.4 GBq per injection, but its response varies from patient to patient. Dosimetry provides the opportunity to personalize treatment, but it requires multiple post-injection images to monitor the radiopharmaceutical’s biodistribution over time. This imposes an additional imaging burden on centers with limited resources. This review explores methods to lessen this burden by optimizing acquisition types and minimizing the number and duration of imaging sessions. After summarizing the different steps of dosimetry and providing examples of dosimetric workflows for $^{177}{\hbox {Lu}}$-DOTATATE and $^{177}{\hbox {Lu}}$-PSMA, we examine dosimetric workflows based on a reduced number of acquisitions, or even just one. We provide a non-exhaustive description of simplified methods and their assumptions, as well as their limitations. Next, we detail the specificities of each normal tissue and tumors, before reviewing dose-response relationships in the literature. In conclusion, we will discuss the current limitations of dosimetric workflows and propose avenues for improvement.

## Introduction

$^{177}{\hbox {Lu}}$-based radiopharmaceutical therapy (RPT) is an emerging treatment modality that has demonstrated additional therapeutic benefits in phase III studies for neuroendocrine tumors ($^{177}{\hbox {Lu}}$-DOTATATE, NETTER-1 trial [1, 2]) and metastatic castration-resistant prostate cancer ($^{177}{\hbox {Lu}}$-PSMA-617, VISION trial [3]) compared to standard systemic treatment. These therapies are standardized with 4 or 6 cycles of 7.4 GBq per cycle, although treatment responses vary among patients. Objective response rates (partial and complete) were 18% and 51% for $^{177}{\hbox {Lu}}$-DOTATATE and $^{177}{\hbox {Lu}}$-PSMA-617, respectively. Implementing individualized dosimetry for all patients would enable personalized treatments, thereby enhancing treatment responses [4]. This has already been demonstrated in the context of radioembolization by Garin et al. [5] for locally advanced hepatocellular carcinomas. Several trials have also been conducted with $^{177}{\hbox {Lu}}$-DOTATATE [6–8]. The authors adjusted either the injected activity per cycle (P-PRRT trial [6]) or the number of cycles (ILUMINET trial [7]) to reach a maximum tolerated dose (MTD) for the kidneys (23 Gy or a biological effective dose of 27 or 40 Gy). This led to an increased response rate with activity adjustment and a higher absorbed dose by the lesions with cycle number adjustment.

*Implementation of dosimetry* In 2024, a European study [9] highlighted significant disparities among countries in the implementation of dosimetry for $^{177}{\hbox {Lu}}$ therapies, with some countries conducting both pre- and post-treatment dosimetry while others perform none. One explanation for this variation is the additional time and resources involved, as estimated by Gabina et al. [10], who found that dosimetry for two lesions and one kidney in $^{177}{\hbox {Lu}}$-DOTATATE therapies takes approximately 8 h, to which an additional 8.7 h must be added for preparing the gamma camera to ensure quantitative imaging. Indeed, the dosimetric process can be divided into three main components: 1) calibration and preparation of measurement tools (such as a dose calibrator and gamma camera) to obtain precise activity quantification, 2) post-injection acquisitions to monitor the radiopharmaceutical biodistribution over time, and 3) data processing to calculate absorbed doses. Currently, there is no standardized procedure for each step, but recommendations have been provided by the EANM Dosimetry Committee [11]. Each center makes choices based on the number of feasible acquisitions, their duration, defined time-points, as well as the patient’s health status and technical and logistical constraints.

*Previous reviews* Several literature reviews have examined various dosimetric workflows implemented by centers based on their resources for $^{177}{\hbox {Lu}}$-DOTATATE [12, 13] and $^{177}{\hbox {Lu}}$-PSMA [14–16] therapies. These reviews discuss dosimetric methods [12, 14] as well as specific aspects of dose calculations for organs at risk (OAR) and tumors [13–16]. Some also provide dose values [13–16] and even description of dosimetric workflows [13, 15]. Most workflows rely on multiple post-injection acquisitions, although it is not always feasible to perform them. None of them address strategies for reducing the imaging burden associated with dosimetry in clinical practice. Only a few EANM guidelines [17], along with examples of dosimetric workflows, have been provided to aid and guide centers in implementing dosimetry according to their resources.

*Goal* This review aims to present dosimetric methods based on a reduced number of post-injection acquisitions, highlighting their advantages, drawbacks, application conditions, and limitations. Additionally, we will discuss the selection of optimal acquisition times and describe the specificities of each volume of interest (VOI) for dosimetry before examining dose-response relationships and dose-toxicity studies necessary for therapy personalization.

## Literature review synthesis

A bibliographic investigation was conducted through PUBMED searches,^1^ utilizing various keywords such as “Dosimetry,” “177Lu-PSMA,” “177Lu,” “177Lu-DOTATATE,” “Single time-point,” “toxicity,” “response,” “review,” etc. The search results underwent manual analysis, and papers were selected based on their content. This process yielded a final selection of 155 articles. The clinical trials related to treatment adaptation through dosimetry have also been investigated and included via ClinicalTrials.gov.^2^ The articles were gathered and classified into several tables, providing a quick synthesis of their content for comparison purposes.

First, Table 1 describes published studies that present or compare dosimetric methods relying on a limited number of acquisitions. The top part of the table, above the dashed line, encompasses studies with approaches relying on a single acquisition, whereas the bottom section comprises those comparing reference methods with approaches requiring a reduced number of acquisitions. Table 1 is associated with Table 2 describes the hypotheses, methods, and effective half-life used for dosimetry based on a single acquisition.

Then, Tables 3 and  4 synthesise published dosimetric workflows for $^{177}{\hbox {Lu}}$-DOTATATE and $^{177}{\hbox {Lu}}$-PSMA therapies. Each table indicates the reference article, the number of patients, the type of images and TP, and some information about the dosimetry method. Ranges of estimated absorbed dose values are also provided as well as effective half-life times when available.

In the following sections, we review the tables contents throughout the different steps of dosimetry, image acquisitions workflow and image-based dosimetry. More details are then given individually for several normal tissues such as kidneys, bone marrow, salivary and lacrimal glands and for tumors, reviewing dose-toxicity and dose-response relationship based on the selected papers.

## Analysis

### Dosimetry in clinical practice

Introducing dosimetry in clinical practice is challenging as it requires additional time and resources, even though it is neither mandatory nor essential for patient treatment currently. Its implementation can be divided into three main steps: (1) calibration and preparation of measurement tools, (2) monitoring the biodistribution of the radiopharmaceutical over time using imaging, and (3) dose estimation through data processing.

#### Calibrating and preparing measurement tools

The initial phase entails calibrating both the activity meter and the gamma camera for a set of acquisition and reconstruction parameters, either provided by the manufacturer or determined following optimization. The activity meter (dose calibrator) guarantees accurate measurement of the administered activity to the patient, while the gamma camera enables the assessment of the radiopharmaceutical’s biodistribution within the patient at a given time. Hence, calibrating the gamma camera is required to convert counts/mL measurements to Bq or Bq/mL for dosimetry purposes. Various methodologies are documented in the literature, with some outlined in MIRD n$^{\circ }$23 [18].

#### Radiopharmaceutical biodistribution monitoring

Monitoring the biodistribution of the radiopharmaceutical within the patient via multiple post-injection acquisitions is crucial for estimating the pharmacokinetic parameters of the volumes of interest. However, the availability of the gamma camera can pose challenges as each acquisition requires additional time and resources, both in terms of personnel and costs. Furthermore, patient’s health statuses may not always permit extended or repeated acquisitions due to discomfort and fatigue. Consequently, each center determines the most appropriate acquisition protocols based on its organizational constraints and the individual patient’s circumstances, as illustrated by Tables 3 and 4.

#### Absorbed dose calculations

Volumes of interest are delineated for each acquisition to assess the cumulative activity within these regions by fitting and integrating the Time Activity Curve (TAC). Subsequently, the absorbed dose is calculated using a dosimetric method.

*Segmentation* The contouring step of the volumes of interest can be time-consuming. Two main approaches are commonly used: contouring for each acquisition [19, 20] or contouring on a single acquisition followed by propagation onto others after registration between them. While the latter method saves time, it relies on the quality of registration. Typically, rigid registration is assumed [6, 21, 22], although the patient may not be in exactly the same position for each acquisition. However, a deformable registration approach [23, 24] can also be considered. There is no clear consensus on the preferred contouring method but this choice impacts absorbed dose estimations [25, 26]. Segmentation based on CT images leverages anatomical information but does not account for partial volume effects, unlike fixed or adaptive thresholding methods used for segmenting SPECT images. This approach is also relevant for dosimetry of small lesions not visible on CT images. However, it is worth noting that the contouring will depend on the corrections applied during reconstruction [27]. A literature review has been proposed by Gawel et al. [28] to compile the different existing methods. Finally, automatic organ segmentation methods based on deep learning have been developed to expedite the process, such as TotalSegmentator [29] or Moose [30] (among open-source methods).

*Time Activity Curve*

The curve fitting step allows modeling the biokinetics of $^{177}{\hbox {Lu}}$ radiopharmaceuticals, which involves a binding phase followed by an elimination phase, further divided into biological elimination (urine) and radioactive decay. Typically, mathematical models such as trapezoidal, linear, mono-exponential, bi-exponential, or tri-exponential models [31] are employed, particularly when the number of Time Points (TP) is limited (refer to Table 2). The selection of the model depends on the organ under consideration and can be adapted for voxel-level analysis. This choice is important as it can influence the estimated absorbed dose, as highlighted by Guerriero et al. [32] for the kidneys when acquisitions were not available up to at least two times the effective half-life. This is why Sarrut et al. [23] used the Akaike criterion to identify the optimal fitting model, which was then integrated into the automated dosimetric workflow proposed by Dewaraja et al. [33]. In Single Time-Point (STP) methods, the mono-exponential model is predominantly favored although Hänscheid et al. [34] demonstrated its suitability in only 25/54 cases for the kidneys, 12/25 for the liver, 3/27 for the spleen, and 7/22 for tumors in their patient cohort (Pearson’s r>0.98).

*Dosimetry methods*

Several dosimetric methods are available for determining the absorbed dose within a volume, including Monte Carlo simulations, the MIRD formalism and S-values, dose kernels, and the local dose deposition method. These methods have been discussed, along with their assumptions, advantages, and limitations, in the review by Huizing et al. [12] To streamline clinical implementation, various dosimetry software packages have been developed and compared [35–38], as they differ in terms of segmentation, fitting model selection, and dosimetric approach. Some methods may require a minimum number of acquisitions for effective utilization. Additionally, automation of the dosimetric workflow, as proposed by Dewaraja et al. [33] (approximately 25 min), can help reduce data processing times.

### Clinical imaging

The main obstacle to implementing dosimetry in clinical practice is the additional time and resources required. Among the three points mentioned earlier, monitoring the biodistribution of the radiopharmaceutical over time is the most problematic. Indeed, calibration and optimization of measurement tools are done intermittently, while data processing is increasingly facilitated by the introduction of new tools and software. Therefore, in the remainder of this review, we will explore ways to reduce the burden of imaging.

#### 2D planar or 3D SPECT acquisition or both

The choice between a post-injection 3D SPECT or 2D planar acquisition will impact the duration of the acquisition. 2D planar acquisitions are quick to perform, especially for whole-body imaging (WB), but they involve organ overlap, which can lead to uncertainties in attenuation correction and segmentation [20]. 3D SPECT acquisitions, on the other hand, are longer, especially when a wide field of view is required: for WB imaging, multiple bed positions are necessary, which can take over an hour for acquisition. However, organs do not overlap in 3D SPECT images and can be segmented using the CT typically acquired concurrently, resulting in more precise attenuation correction and the ability to discriminate between components of activity overlaid along the anterior-posterior axis. This choice significantly affects dosimetric estimates, as demonstrated by Willowson et al. for $^{177}{\hbox {Lu}}$-DOTATATE, where kidney dose was three times higher with 2D planar acquisitions compared to 3D SPECT acquisitions [39]. Currently, 3D SPECT acquisitions are recommended for precise quantification with $^{177}{\hbox {Lu}}$, although it is not always feasible to perform them after each injection. Therefore, a hybrid method, combining multiple 2D planar acquisitions with one 3D SPECT acquisition, may be preferred. Additionally, it provides more accurate results (closer to 3D SPECT acquisitions) than the 2D planar method [20] and is commonly used for therapies involving $^{177}{\hbox {Lu}}$-PSMA-RTL, as patients often have lesions distributed throughout the body, requiring whole-body imaging. In Table 4, only 7 out of 28 studies are based on 3D SPECT acquisitions involving multiple bed positions.

#### 360$^\circ$ CZT gamma camera

Recently, the introduction of 360$^\circ$ CZT gamma cameras, equipped with twelve detection heads, has enabled faster acquisitions for whole-body 3D SPECT images. Currently, there are two commercially available cameras: StarGuide (General Electric Healthcare, Haifa, Israel) and VERITON (Spectrum Dynamics, Caesarea, Israel).

A study conducted by Song et al. [40] compared the performance of the StarGuide camera to an Anger-type gamma camera (GE Discovery 670 Pro) for $^{177}{\hbox {Lu}}$ therapy. The results showed comparable detection rates between the two cameras, while the acquisition time was three times shorter for the CZT camera (12 min vs. 32 min). Similarly, another study [41] evaluated VERITON with a Symbia camera (3/8” NaI thickness) for four different radionuclides ($^{99m}{\hbox {Tc}}$, $^{123}{\hbox {I}}$, $^{201}{\hbox {Tl}}$, and $^{111}{\hbox {In}}$) and demonstrated the potential to reduce the acquisition time by a factor of 2 to 3 for the VERITON camera while maintaining the same image quality.

The quantitative performance of the VERITON camera for $^{177}{\hbox {Lu}}$ was investigated by Vergnaud et al. [26], who proposed an initial set of reconstruction parameters, and a similar study was suggested by Danieli et al. [42] for the StarGuide camera. Furthermore, a clinical trial (NCT04467567, EVADOVE177Lu) is currently in progress to compare the dosimetry obtained from an Anger-type gamma camera with that from a CZT gamma camera.

### How many acquisitions?

The number of post-injection acquisitions in dosimetry presents two major challenges: the precision of estimation and the burden of imaging for both the patient (returning to the hospital, undergoing multiple acquisitions) and the hospital service (requiring additional time and financial resources). To facilitate its implementation into clinical practice, reducing the number of acquisitions is being considered. The strategies include predicting the total absorbed dose from the doses of the initial cycles, reducing the number of acquisitions, and performing dosimetry based on a single acquisition.

#### Time-point reduction and selection

*Absorbed dose prediction*

The prediction of absorbed dose, based on dosimetry from either the initial cycle or the first two cycles, offers a means to reduce the number of acquisitions in subsequent cycles. Two approaches have been proposed in the literature. Firstly, the cumulative absorbed dose is estimated based on the absorbed dose per administered activity in the initial cycle, with potential precision enhancement through additional acquisitions after certain subsequent cycles (e.g., Mix et al. [43] for $^{177}{\hbox {Lu}}$-PSMA in kidneys and Pirozzi Palmese et al. [44] for $^{177}{\hbox {Lu}}$-DOTATATE). This approach has been supported by studies such as that conducted by Pirozzi Palmese et al. [44], which demonstrated significant precision enhancement by incorporating dose estimation at cycle 4 for kidneys, liver, spleen, bone marrow, and lesions. The second approach aims to predict whether the cumulative absorbed dose will exceed a safety threshold for the kidneys (25 Gy), based on the absorbed dose(s) after the initial cycle(s). An algorithm has been provided by Chicheportiche et al. [45] to determine if additional acquisitions are necessary for prediction based on the absorbed dose after the first cycle.

*Reduction*

The optimization of the number of acquisitions after each cycle has been studied to determine the preferred TPs to maintain the highest possible accuracy.

In the context of $^{177}{\hbox {Lu}}$-DOTATATE therapies, early acquisitions are discouraged to avoid biasing dose estimates (<24 h [46] or <48 h [47]), while late acquisitions are necessary [48] and should be performed at least twice the effective half-life time [32]. These considerations have been confirmed when reducing acquisitions in several protocols, where the ideal TPs were 24 h and 168 h [19, 49–51] or 24 h and 72 h [52]. Sundlöv et al. [53] compared different combinations of times and image types, demonstrating that using a single SPECT acquisition at 96 h was the most accurate method. Sometimes, the number and timing of acquisitions are constrained by clinical circumstances, and not all acquisitions can always be performed due to the patient’s health status or technical issues. Hence, Vergnaud et al. [54] proposed an algorithm to calculate absorbed dose from available data. One approach involves conducting precise dosimetry after the first cycle to determine the patient-specific effective half-life time, which can then be used for subsequent cycles when only a 24-h acquisition is performed [55]. It should be noted that the optimal acquisition times determined by Peterson et al. [47] depend on the organ studied: 3–5 days for a single TP, except for the spleen (6–8 days), 1–2 days and 3–5 days for two TPs, and 1–2 days, 3–5 days, and 6–8 days for three TPs. Therefore, the optimal time for the kidneys is not necessarily the same for all the OARs, or lesions.

A limited number of studies are available for $^{177}{\hbox {Lu}}$-PSMA therapies. For kidneys [22, 56] and salivary glands [57, 58], a single TP at 48 h appears optimal for dose estimation, with the potential to improve precision by adding a late acquisition (168 h [58]). Conversely for lesions, the opposite is true: a late TP is necessary while the early TP enhances estimation precision. The compromise for kidney and tumor dosimetry is based on two TPs at 20 h and 192 h for Rinscheid et al. [56] and at 20–60 h and 40–200 h for Chen et al. [59]. The same TPs were selected for bone marrow by Grob et al. [60] (1d and 7d). Finally, if there are three acquisitions, time points at days 1, 3, and 7 should be prioritized but could be replaced by days 1, 2 and 3 for logistical reasons [61].

#### Single time-point methods (STP)

Single time-point methods for estimating Time-Integrated Activity (TIA) are increasingly studied for $^{177}{\hbox {Lu}}$ therapies. Using a single acquisition does not allow access to the radiopharmaceutical biodistribution over time, which is essential for estimating absorbed doses. Therefore, simplification [62], prior information [34, 63–66], or even a model [67–71] is required. Details are provided in Tables 1 and 2.

Hänscheid et al. [62] and Madsen et al. [65] proposed STP methods using a mono-exponential function to fit the time-activity curve (TAC) and estimated cumulative activity from a theoretical approximation or by using the effective half-life of a population of patients, respectively. Hou et al. [72] compared these two methods for various radiopharmaceuticals ($^{177}{\hbox {Lu}}$-DOTATATE, $^{90}{\hbox {Y}}$-DOTATOC, $^{177}{\hbox {Lu}}$-PSMA-617 et $^{177}{\hbox {Lu}}$-PSMA-I &T) and showed that errors compared to the reference protocol can be large except for $^{177}{\hbox {Lu}}$-DOTATATE and kidney dosimetry. The validity of these methods was studied by Gustafsson et al. [73], showing that it depends on inter-patient variability, the relationship between biological and physical decay constants, and the acquired TP, with a risk of underestimating the cumulative activity with STP methods. The method proposed by Madsen et al. [65] was also evaluated by Zhao et al. [64], who found that the majority of cumulative activity was between 1 day and infinity for $^{177}{\hbox {Lu}}$-DOTATATE therapy. It is possible to limit the error (<10% in 98% of cases) by choosing a TP between $T_{eff}$ and 1.5$T_{eff}$ of the patient population. However, using the pharmacokinetic parameters of the patient at cycle 1 for subsequent cycles [63] is recommended by Willowson et al. [55] as opposed to those of a patient population.

Comparing methods and determining corresponding optimal TPs help reduce uncertainties but, acquisitions cannot always be performed at these optimal times. Therefore, Wang et al. [69] proposed data-driven models for $^{177}{\hbox {Lu}}$-DOTATATE treatments to reduce the sensitivity of estimates to the choice of TPs, notably by including biomarkers for early acquisitions. This approach allows acquisitions to be conducted based on clinical conditions rather than the optimal TP for each method. Generally, a late acquisition time is recommended (>72 h), although the activity is low and the quantification accuracy may be compromised.

Various simple mathematical functions are commonly used to fit time-activity curves (TAC), as described in Tables 3 and 4. Some effects are common across all patients (known as fixed effects), while others are specific to each individual (known as random effects) and can be effectively described using non-linear mixed models (NLMM). In a study by Devasia et al. [68], the cumulative kidney activities estimated from a single TP were compared using NLMM and the mathematical models proposed by Hänscheid et al. [62] and Madsen et al. [65] for $^{177}{\hbox {Lu}}$-DOTATATE therapies. The results showed that, on average, the NLMM approach exhibited lower bias and fewer outliers compared to the mathematical models. The inclusion of a physiologically-based pharmacokinetic (PBPK) model [70] did not alter the estimates of cumulative activity compared to the NLMM model alone, and the differences in absorbed doses for the kidneys and tumors were not statistically significant ($^{111}{\hbox {In}}$-DOTATATE). The NLMM model was also associated with a population-based model selection to improve fit selection and was tested for kidney dosimetry during $^{177}{\hbox {Lu}}$-PSMA therapy. The doses were accurate when the acquisition was performed at 2 days.

The choice of method depends on the most important information: the absorbed dose by organs at risk, that by tumors, or the preservation of patient management (choice of the number of administered cycles and the number of acquisitions to be performed after each cycle). For example, Chicheportiche et al. [67] proposed a trained multiple linear regression model to estimate the dose from acquisition at 168 h after cycle 1 and at 24 h after subsequent cycles, which was validated on a cohort of 159 patients [74] treated with $^{177}{\hbox {Lu}}$-DOTATATE. They demonstrated that the number of administered cycles, the number of acquisitions per cycle for each patient, and the doses were similar to the multiple time-point method.

**Table 1:** Description of studies that present or compare dosimetric methods relying on a limited number of acquisitions

Authors	Treatment	Number of	Acquisitions	Reference	Single or Reduced	Volumes	Curve fit
		patients		Time-Points	Time-Points tested		
Garske et al. (2012) [63]	$^{177}{\hbox {Lu}}$-DOTATATE	30 NETs	SPECT/CT	24 h, 72 h, and 168 h	24 h (STP)	K, L, and S	Mono-exp
Hänscheid et al. (2017) [34]	$^{177}{\hbox {Lu}}$-DOTATATE/TOC	21 = NETs and	Planar acquisitions	1–4 h, 1 d, , and $\ge$ 4d	24 h or 48 h or 72 h (STP)	K, T, L, and S	Mono- or bi-exp
		8 = meningioma					
Hänscheid et al. (2018) [62]	$^{177}{\hbox {Lu}}$-DOTATATE/TOC	21 = NETs and	Planar acquisitions	At least: 1–4 h, 1 d, 2 d, and $\ge$ 4 d	24 h or 48 h or 72 h or 96 h or	K, T, L, and S	Mono- or bi-exp
		8 = meningioma			120 h or 144 h (STP)		
Zhao et al. (2019) [64]	$^{177}{\hbox {Lu}}$-DOTATATE	39 NETs	SPECT/CT	4 h, 23 h, and 70 h	4.3 h or 22.7 h or 69.8 h (STP)	K	Trapezoidal + mono-exp
Chicheportiche et al.	$^{177}{\hbox {Lu}}$-DOTATATE	72 metastatic NETs	SPECT/CT	24 h, 96 h, and 168 h then 24 h	24 h or 96 h or 168 h (STP)	K, BM, L, S, and T	Mono-exp
(2021) [67]							
Devasia et al. (2021) [68]	$^{177}{\hbox {Lu}}$-DOTATATE	10 NETs (250 virtual)	SPECT/CT	Up to 4 TPs	96h (STP) or 4 h and 96 h	K	NonLinear Mixed
				including one at 96h			Model (NLMM)
Wang et al. (2023) [69]	$^{177}{\hbox {Lu}}$-DOTATATE	27	SPECT/CT	3–5 h, 1–2 d, 3–5 d, and 6–8 d	3–5 h or 23–51 h or 72–126 h	K and T	Bi-exp
					or 144–193 h (STP)		
Madsen et al. (2019) [65]	$^{90}{{\hbox {Y}}}$-DOTATATE	NA	SPECT/CT	30 min, 24 h, 48 h, and 72 h	5 h or 24 h or 48 h or	K	Mono- or bi-exp
					72 h (STP)		
Hardiansyah et al.	$^{111}{{\hbox {In}}}$-DOTATATE	4 = NETs	Planar acquisitions	2 h, 4 h, 24 h, 48 h,	2 h or 4 h or 24 h or 48 h	K and T	NonLinear Mixed
		4 = meningioma		and 72 h	or 72 h (STP)		Effect model (NLME)
(2022) [70]							
Jackson et al. (2020) [66]	$^{177}{\hbox {Lu}}$-PSMA-617	30 mCRPC	SPECT/CT	4 h, 24 h, and 96 h	All hours between 5 h and	T, PG, SubG, K, L, and S	Tri-exp
					149 h		
Hardiansyah et al.	$^{177}{\hbox {Lu}}$-PSMA-617	63 mCRPC	SPECT/CT	1.8 h, 18.7 h, 42.6 h,	1.8 h or 18.7 h or 42.6 h	K	Sum of
				66.3 h, and 160.3 h	or 66.3 h or 160.3 h		Exponentials
(2024) [71]							
Larsson et al. (2012) [48]	$^{177}{\hbox {Lu}}$-DOTATATE	33 NETs	Planar acquisitions	1 h, 1 d, 2 d, and 7 d	Exclusion of each TP	K	Mono-exp
					successively		
Guerriero et al. (2013) [32]	$^{177}{\hbox {Lu}}$-DOTATATE	28	SPECT/CT	2 h, 6 h, 20 h,	Excluding the 6-h	K	Seven diffe-
				44h, and 67h	or 3-day acquisitions		rent methods
Delker et al. (2015) [46]	$^{177}{\hbox {Lu}}$-DOTATATE	64 metastatic NETs	Planar acquisitions	1 h, 24 h, 48 h,	Excluding the 1-h	K	Linear +
				and 72 h	acquisition		Bi-exp
Heikkonen et al. (2016) [19]	$^{177}{\hbox {Lu}}$-DOTATATE	24	SPECT/CT	24 h, 72 h, and 168 h	24 h or 72 h or 168 h	K	Mono-exp
					or 24h and 168h		
Del Prete et al. (2018) [52]	$^{177}{\hbox {Lu}}$-DOTATATE	79 NETs	SPECT/CT	4 h, 24 h, and 72 h	1 d and 3 d or 3 d (all	K, BM,and T	Mono-exp
					cycles) or 1 d and 3 d (C1)		
					With 1 d or with 3 d or		
					Without (others cycles)		
Sundlöv et al. (2018) [53]	$^{177}{\hbox {Lu}}$-DOTATATE	22	Planar acquisitions	1 h, 24 h, 48 h or 96 h	24 h or 96 h	K	Mono-exp
			+ one SPECT/CT	and 168 h + 24 h			
Willowson et al. (2018) [55]	$^{177}{\hbox {Lu}}$-DOTATATE	18	SPECT/CT	4 h, 24 h, and 96–120 h	4 h or 24 h	K	Mono-exp
Chicheportiche et al.	$^{177}{\hbox {Lu}}$-DOTATATE	25 metastatic NETs	SPECT/CT	24 h, 96 h, and 168 h	24 h and 96 h or 24 h and	K, BM, L, S, and T	Mono-exp
(2020) [49]					168h or 96h and 168h		
Freedman et al. (2020) [50]	$^{177}{\hbox {Lu}}$-DOTATATE	30 NETs	SPECT/CT	4 h, 24 h, 4–5 d, and 1 w	24 h and 4–5 d and 1 w or 24 h	K, L, S, and T	Mono-exp with or
					and 1 w or 24 h and 4–5 d or		Without trapezoidal rule
					4–5 d and 1 w		
Sandström et al. (2020) [51]	$^{177}{\hbox {Lu}}$-DOTATATE	777	SPECT/CT	1 d, 4 d, and 1 w	1 d and 4 d or 1 d and 7 d	K	Mono-exp
					or 4 d		

Authors	Treatment	Number of	Acquisitions	Reference	Single or Reduced	Volumes	Curve fit
		patients		Time-Points	Time-Points tested		
Vergnaud et al. (2022) [54]	$^{177}{\hbox {Lu}}$-DOTATATE	20 NETs	SPECT/CT	1 h, 24 h, and 96–144 h	1 h or 24 h or 96–144 h	K, L, S, and BM	Tri-exp
				6 h, 24 h, and 1 w			
Chicheportiche et al.	$^{177}{\hbox {Lu}}$-DOTATATE	159 NETs	SPECT/CT	24 h, 96 h, and 168 h	168 h for C1 and 24h for	K, BM, L, S, and T	Mono-exp
(2023) [74]					subsequent cycles		
Peterson et al. (2023) [47]	$^{177}{\hbox {Lu}}$-DOTATATE	28	SPECT/CT	4 h, 24 h, 96 h, and 168 h	4 h or 24 h or 96 h or 168 h	LK, RK, L, S, 5 T	Mono- or Biexp
					or combinations of 2 or 3 TPs		
Gosewisch et al.	$^{177}{\hbox {Lu}}$-DOTATATE	5 NETs	Planar acquisitions	24 h, 48 h, and 72 h	24 h or 48 h or 72 h for	BM	Mono-exp
(2018) [75]	$^{177}{\hbox {Lu}}$-PSMA-617	5 mCRPC	+ SPECT/CT		Planar acquisition +		
					3 SPECT/CT		
Kurth et al. (2021) [57]	$^{177}{\hbox {Lu}}$-PSMA-617	46	SPECT/CT	2 h, 24 h, 48 h,	24 h or 48 h or 72 h	K and PG	Bi-exp
			Planar acquisitions	and 72h			
Brosch-Lenz et al.	$^{177}{\hbox {Lu}}$-PSMA-617	20 mCRPC	SPECT/CT	24 h, 48 h, and 72 h	24 h or 48 h or 72 h	K and T	Mono-exp
(2023) [22]							
Chen et al. (2023) [59]	$^{177}{\hbox {Lu}}$-PSMA-617	18 mCRPC	SPECT/CT	2 h, 20 h, 40 h,	40 h or 60 h or 200 h	K and T	Mono-exp
				60 h or 200 h	or 2 h+20 h or		
					2 h+40 h or 2 h+60 h or		
					2 h+200 h or 20 h+40 h		
					or 20 h+60 h or 20 h+200 h or		
					40 h+60 h or 40 h+200 h		
Peters et al. (2023) [58]	$^{177}{\hbox {Lu}}$-PSMA-617	10 HSPC	SPECT/CT	1 h, 24 h, 48 h,	1 h or 24 h or 48 h or 72 h or	K, L, SG,	Mono-exp
			Planar	72 h, and 168 h	168 h or 1 h+24 h or 1 h+48 h	and T	
					or 1 h+72 h or 1 h+168 h or		
					24 h+48 h or 24 h+72 h or		
					24 h+168 h or 48 h+72 h or		
					48 h+168 h or 72 h+168 h		
Grob et al. (2024) [60]	$^{177}{\hbox {Lu}}$-PSMA-617	8 mHSPC	SPECT/CT	1 h, 1 d, 2 d,	2 d and 7 d	BM	Mono-exp
				3 d, and 7 d			
Rinscheid et al. (2019) [76]	$^{177}{\hbox {Lu}}$-PSMA-I &T	13 mCRPC	Planar acquisitions	Between 1 h	2640 sampling schedules	K	Mono-exp
	for simulations		+ one SPECT	and 192h	were investigated		
Rinscheid et al. (2020) [56]	$^{177}{\hbox {Lu}}$-PSMA-I &T	13 mCRPC	Planar acquisitions	Between 1 h	276 sampling schedules	K and T	Mono-exp
	for simulations		+ one SPECT	and 192h	with 2 TPs, 2024 with		
					3 TPs, and 5700 with 4 TPs		
Resch et al. (2023) [61]	$^{177}{\hbox {Lu}}$-PSMA-I &T	5	SPECT/CT	1 d, 2 d, 3 d, and 1 w	1 d and 2 d and 3 d or	K and T	Mono- or bi-exp
					2 d and 3 d and 7 d or		
					1 d and 2 d and 7 d or		
					1 d and 3 d and 7 d or		
					1 d and 2 d or 1 d and		
					3 d or 1 d and 7 d		
Maaß et al. (2016) [77]	$^{111}{{\hbox {In}}}$-DTPAOC	15 NET	Planar acquisitions	0.75 h, 5 h, 1 d,	Omission of 1 or 2 or	K, L, S, T,	Sum of
				2d, and 3 or 5d	3 or 4 TP(s)	serum, and WB	exponentials

The top section of the table encompasses studies introducing approaches relying on a single acquisition, whereas the bottom section comprises those comparing reference methods with approaches requiring a reduced number of acquisitions

K: Kidneys, L: Liver, S: Spleen, T: Tumor, NET: neuroendocrine tumor, SubG: Submandibular Gland, PG: Parotid Gland, SG: Salivary glands, NA: Not Available, d: day, w: week, C1: cycle 1

**Table 2:** Hypotheses, methods, and effective half-life for dosimetry based on a single acquisition

Authors	Hypothesis	Methods	Effective Half-lives Used
Garske et al. (2012) [63]	Unchanged effective half-life	Mono-exp fit with a reuse of	K for cycles 1 and 4
	between cycles	effective half-life from cycle 1	[43.1h, 76.3h]
Hänscheid et al. (2017) [34]	Fixed tissue specific half-lives	Mono-exp fit with empirical	Values used: K: 50h, L and
	If bi-exp fit, approximation by the longer	Effective half-lives	S: 67h, NET: 77h
	effective half-life and a mono-exp fit		
Hänscheid et al. (2018) [62]	$2^{ \frac{t_{l}}{T_{eff}}} \times T_{eff}$ approximated by $2 \times t_{l}$	Approximation of the integral	Same patients as [34]
		(cumulative activity) from	
		a TP at 4d	
Zhao et al. (2019) [64]	Population mean effective	Mono-exp fit + population	K: 45h (mean)
	half-life	mean effective half-life	
Chicheportiche et al. (2021) [67]	Pharmacokinetic curves	Trained Multiple Linear Regression model	NA
	from a population of	(MLR) + acquisition at 168h (cycle 1)	
	patients	or at 24h (other cycles)	
Devasia et al. (2021) [68]	Time-activity data of a	Nonlinear mixed model	K: 52h
	historical group of patients	+ one or two acquisition(s)	
Wang et al. (2023) [69]	Biexponential time-activity	Generalized additive	Slowest component of
	curves	models (GAM)	the biexp fit T: 89.5±35.5h
			LK: 51.7±13.4 h / RK: 50.3±14.4h
			K: 51.2 ± 13.7 h
Madsen et al. (2019) [65]	Prior information of	Population kinetic parameters	NA
	kinetic parameters from a	+ one acquisition	
	population of patients		
Hardiansyah et al. (2022) [70]	Prior knowledge about	PBPK model + NLME +	NA
	a population of patients	acquisition at 47h	
Jackson et al. (2020) [66]	Homogeneity of pharmacokinetic	Tissue-specific dose	NA
	parameters	conversion factors for	
		common posttreatment imaging	
		times	
Hardiansyah et al. (2024) [71]	Prior knowledge about	NLME model +	Two clearance phases:
	a popultation of patients	PBMS + acquisition at 2d	11.5 h and 76.4 h

PBPK: Physiologically Based Pharmacokinetic Model, PBMS: Population-Based Model Selection

#### Limitations of simplified methodologies

The simplified dosimetry methods have limitations. Firstly, some have only been evaluated and compared for kidney dosimetry [64, 65, 68, 71], while other OARs and tumors should also be considered.

Secondly, each study has its own reference protocol with specific imaging types, a certain number of TPs, and specific TPs. Therefore, the estimated optimal TPs are only optimal relative to the available TPs. This is demonstrated by Peterson et al. [47], who compared optimal TPs obtained from clinical and simulated data. Additionally, the methods have not been evaluated for all radiopharmaceuticals; they are primarily evaluated for $^{177}{\hbox {Lu}}$-DOTATATE (Tables 1 and 2). Results obtained for one radiopharmaceutical cannot be directly applied to another, as shown by Hou et al. [72].

Thirdly, some simplified methods only estimate absorbed dose from self-irradiation [34, 62], while certain VOIs may receive cross-irradiation from nearby organs and tumors with high uptake. Hence, the estimated absorbed dose does not reflect the total absorbed dose accurately.

Fourthly, the effective half-life may vary from one cycle to another [22, 63] due to treatment efficacy (tumor sink effect) or tumor progression. Consequently, reusing it may result in errors in absorbed dose estimation. Unfortunately, this method has not been extensively compared in the literature. Furthermore, the results can be influenced by using an effective half-life derived from a patient population, as there is inter-patient variability, especially in lesion kinetics [78]. This method assumes that the patient’s pharmacokinetics are similar to those of the cohort. It may be relevant to establish criteria to identify patients who do not meet this condition. In such cases, additional acquisitions may be recommended. Finally, this approach necessitates having a cohort of patients with multiple post-treatment acquisitions, representing the diversity of treated patients. Conversion factors have been provided by Jackson et al. [66] for $^{177}{\hbox {Lu}}$-PSMA, but they are not available for all tissues, particularly bone marrow due to the presence of infiltrating lesions.

Fifthly, each hospital has its own schedule for post-injection acquisitions, which may not always be performed due to the patient’s health status, technical limitations, or logistical reasons. For instance, this may be the case for late acquisition, which is generally recommended to minimize estimation errors [62, 72], especially when patients leave the hospital after 24 h (France). Consequently, dosimetry methods may vary from one cycle to another, impacting the errors in absorbed dose estimation. When STP methods are used, the cumulative activity is generally underestimated [57]. Moreover, optimal TPs often depend on the method and the tissue considered. To address this issue, Wang et al. [69] developed a TIA estimation model that is less sensitive to the choice of time TP.

Finally, simplified methods have been evaluated under specific conditions and should be independently validated to assess their applicability, as suggested by Hänscheid et al. [79].

### Clinical dosimetry of normal tissues and tumors

Besides the choice of dosimetric method, several factors can influence the accuracy of dose estimates, including segmentation, radiopharmaceutical biodistribution, and image corrections. This section aims to enumerate and describe the specificities of each VOI, such as radiopharmaceutical uptake and localization difficulties.

#### Kidneys

The kidneys filter the blood and are considered OAR for $^{177}{\hbox {Lu}}$ therapies because there is a potential risk of reabsorption with $^{177}{\hbox {Lu}}$-SSTR, as well as PSMA receptors to consider with $^{177}{\hbox {Lu}}$-PSMA. Various risk factors for renal toxicity have been identified, including age, preexisting kidney diseases, diabetes mellitus, and hypertension, particularly in patients undergoing $^{177}{\hbox {Lu}}$-DOTATATE [80] and $^{177}{\hbox {Lu}}$-PSMA-617 [81] treatments. This organ has been considered as a dose-limiting organ [82], with the average absorbed dose used to personalize $^{177}{\hbox {Lu}}$-DOTATATE therapies in several studies (Phase II trials P-PRRT NCT02754297 [6], ILUMINET NCT01456078 [7], or the study [8]) or to evaluate simplified dosimetry methods (Table 1). However, this average absorbed dose does not reflect the heterogeneity of the radiopharmaceutical biodistribution (PRRT), as demonstrated by Konijnenberg et al. [83] through ex-vivo autoradiography of healthy kidney tissue, and the variation in absorbed dose between different regions of the organ. Heikkonen et al. [19] highlighted the impact of contour selection on dosimetry, revealing a 1.7-fold difference between anatomical contouring and contouring based on 4 cm$^{3}$ spheres. These factors can significantly influence the relationship between the absorbed dose by the kidneys and the risk of renal toxicity.

#### Active bone marrow

Bone marrow is a hematopoietic organ, consisting of red marrow (active) responsible for the production of blood cells (red blood cells, white blood cells, and platelets) and yellow marrow, which produces connective tissues in the body, is located within the bones. The distribution and proportion of red marrow depend on the age and gender of the patient [84] (for example, in a healthy adult [85]), making the estimation of absorbed dose by this organ a challenging task due to limited image resolution. Hereafter, active bone marrow will be referred to as bone marrow (BM).

Three main methods have been proposed to estimate the absorbed dose by this organ: biopsy [86], blood samples, and imaging. The first two approaches are invasive and only allow estimation of the self-dose received by the bone marrow. In the case of blood samples, it is assumed that there is no radiopharmaceutical uptake by the bone marrow [87], leading to an underestimation of the absorbed dose during $^{177}{\hbox {Lu}}$-DOTATATE therapies as prolonged elimination of specific uptake has not been considered [88]. Imaging performed for OAR dosimetry can be used to estimate the total absorbed dose to the bone marrow, including the non-negligible cross-dose [89]. A surrogate for the bone marrow is selected near organs with high uptakes, where the absorbed dose is expected to be the highest. Vertebrae are commonly chosen ($^{177}{\hbox {Lu}}$-DOTATATE [90]; $^{177}{\hbox {Lu}}$-PSMA-617 [60]), although the bone marrow is contained within the spongy bone, so the estimated absorbed dose corresponds to both the bone marrow and the spongy bone. Furthermore, dosimetry in this region can be challenging due to bone metastases: 1. they may replace the bone marrow, making the surrogate unusable, and 2. they can significantly irradiate the bone marrow if they are nearby [91]. It is worth noting that a reduction in bone marrow may have been caused by previous treatments. Therefore, accurate localization of the remaining patient’s bone marrow is essential, and implementing voxel-scale dosimetry could enhance the accuracy of these results. Several authors have explored methods to determine the location of bone marrow, including the use of [18F]FLT [92], $^{99m}{{\hbox {Tc}}}$ sulfur colloid, or even $^{99m}{{\hbox {Tc}}}$-anti-granulocyte antibody [93]

#### Salivary glands

The salivary glands are normal organs that can receive high irradiation during $^{177}{\hbox {Lu}}$-PSMA therapies due to the presence of PSMA receptors on which the radiopharmaceutical binds [94]. The toxicities of the salivary glands remain mild to moderate in the studies listed by Mahajan et al. [95]. Several strategies have been investigated to reduce their absorbed dose and thus, the adverse effects. For instance, co-administration of polyglutamate tablets [96] or external cooling [97] have been tested. Reviews summarizing the mechanisms underlying salivary gland toxicity and protective strategies have been compiled by Heynickx et al. [98] and Mahajan et al. [95]. A recent study by Siebinga et al. [99] demonstrated that the salivary glands could reach saturation as the injected activity increased, unlike tumors, where the absorbed dose increases as the injected activity increases (from 3 GBq to 6 GBq injected). This finding could have implications for treatment personalization. Segmentation of the salivary gland volumes is typically performed through direct contouring on the CT images or by incorporating a margin of 1 or 2 cm to account for the partial volume effect, as proposed by Violet et al. [21]. SPECT thresholding also works quite well as these organs are isolated and have quite uniform uptake. This also avoid SPECT-CT misregistration issue which is quite high for the head due to motion (rotation movement).

#### Lacrimal glands

The lacrimal glands, with mean volumes of 0.770 cm$^{3}$ and 0.684 cm$^{3}$ for the right and left glands, respectively [100], are relatively small organs considered dose-limiting in $^{177}{\hbox {Lu}}$-PSMA treatments [101]. This can present challenges in their segmentation. Contouring methods for the lacrimal glands are similar to those used for the salivary glands, although their application can be more challenging due to their small volume in CT images and the low spatial resolution of gamma cameras.

#### Other organs

Other organs are typically monitored dosimetrically, such as the liver and spleen, due to physiological uptake of the radiopeptide [102] in $^{177}{\hbox {Lu}}$-DOTATATE therapy. Blood samples may be collected to monitor liver function (total bilirubin, ALT, AST, GGT, and albumin) [103]. Liver dosimetry can be complicated by the presence of hepatic metastases that are challenging to separate from healthy liver tissue [44]. On the other hand, the spleen, is as a reservoir for blood cells [104] and a filter, absorbing the highest dose among the OARs in $^{177}{\hbox {Lu}}$-DOTATATE therapy (Table 3), which is not the case with $^{177}{\hbox {Lu}}$-PSMA therapy (Table 4). For both organs, contouring is typically anatomical, performed on CT images [105, 106].

#### Tumors

*Dosimetry*

Tumor dosimetry is often conducted to assess whether there is a relationship with treatment response, which could assist in therapy personalization. Two contouring strategies are commonly used: either focusing on individual tumors [107–109], or considering the total tumor volume as a whole [21]. In the first case, only visible and contourable lesions are taken into account, which may not provide a comprehensive representation of the patient’s entire disease. This approach cannot always be combined with biological indicators of disease progression, such as PSA levels in $^{177}{\hbox {Lu}}$-PSMA therapy, which are influenced by the overall tumor burden. On the other hand, the second method allows for such integration, but it can be challenging to implement as it often requires thresholding of functional images, which typically have a limited FOV. Typically, the Total Metabolic Tumor Volume (TMTV) is used, defined as the region with high metabolic uptake after subtracting physiological uptake [21]. Patients may present with multiple types of lesions (e.g., bone, lymph nodes, visceral), each exhibiting heterogeneous radiopharmaceutical uptake [110]. It is essential to consider the heterogeneous density of bone lesions when estimating dosimetry [111], as the characteristics of each lesion can impact the irradiation pattern and, consequently, therapy personalization. Jahn et al. [112] demonstrated that blood perfusion of pancreatic lesions treated with $^{177}{\hbox {Lu}}$-DOTATATE was higher during early cycles than during late cycles, resulting in decrease irradiation over time. Monitoring the uptake evolution over cycles for each lesion type, considering their specific characteristics, could be relevant. Tables 3 to 4 highlight significant differences in absorbed dose between different tumor types. Additionally, Mileva et al. [113] observed a decrease in tumor volume expressing SSTR receptors and a reduction in absorbed dose by gastroenteropancreatic neuroendocrine tumors after the first cycle, both of which are predictive of treatment response.

Finally, some authors have studied the correlation between pre-treatment PET uptake and absorbed dose of lesions (SUV$_{max}$ vs lesion absorbed dose [78]; SUV$_{mean}$ of WB tumor vs absorbed dose of WB tumor [21] in $^{177}{\hbox {Lu}}$-PSMA therapy). However, $^{68}{\hbox {Ga}}$ has a short half-life (67.8 min) compared to biological pharmacokinetics, and the PET acquisition is performed 1 h after injection, which is probably too early to estimate which lesions will be treated. The uptake in the early hours may not necessarily be representative of the dose that will be absorbed during therapy, as it can be followed by a rapid washout. This is why Rosar et al. [114, 115] replaced $^{68}{\hbox {Ga}}$ with $^{89}{{\hbox {Zr}}}$ (t$_{\frac{1}{2}}$= 78.4 h), which enabled later imaging and showed better detection results for malignant prostate tumors not detected with $^{68}{\hbox {Ga}}$ PET in patients with low PSA levels and biochemical recurrence of prostate cancer. Currently, there appears to be no study correlating the PET uptake of $^{89}{{\hbox {Zr}}}$ with the absorbed dose during $^{177}{\hbox {Lu}}$ therapy. $^{64}{\hbox {Cu}}$ (t$_{\frac{1}{2}}$=12.7 h) has also been considered, but less radiochemical stability has been reported [116].

*Tumor Sink Effect* The tumor sink effect refers to increased uptake of the radiopharmaceutical by tumors, with a corresponding decrease in uptake in healthy tissue. A retrospective study including 33 patients demonstrated this effect in the salivary glands, spleen and, potentially the liver [117]. Similarly, Filss et al. [118] observed lower absorbed doses in the salivary glands and kidneys when there was a higher tumor burden in $^{177}{\hbox {Lu}}$-PSMA therapy. Understanding this effect is essential for personalising treatments, especially in cases of extensive disease [16], as it would allow preserving healthy tissues while increasing the absorbed doses to tumors. Hence, Tuncel et al. [119] attempted to identify factors predictive of tumor sink effect and identified three factors: total lesion index uptake on the $^{68}{\hbox {Ga}}$-PSMA PET scan, pre-treatment PSA level, and the rate of change of PSA. They also found that this effect was present in only around a quarter of their patients (17/65).

### Dose relationships

The investigation of dose-response relationships may facilitate personalized treatments to improve patient responses. A literature review has already been published by Cremonesi et al. [120] reporting correlations between absorbed doses, toxicity, and tumor response in PRRT therapies using $^{177}{\hbox {Lu}}$ and $^{90}{{\hbox {Y}}}$.

*Dose-toxicity relationship*

RPT with $^{177}{\hbox {Lu}}$ can cause adverse effects, the toxicity of which is predominantly grades 1 or 2. Grade 3 toxicities affected less than 10% of patients included in the NETTER-1 [1, 2] and VISION [3] trials, except anemia for $^{177}{\hbox {Lu}}$-PSMA (12.9%). Toxicity may increase with higher injected activity if therapy is personalized. Therefore, identifying relationships between the dose absorbed by healthy tissues and toxicities would allow for anticipation and limitation of the risk of grade 3–4 toxicity. Renal and hematological toxicities are the most extensively studied, as they are inherent in all $^{177}{\hbox {Lu}}$ therapies and are monitored through regular blood samplings. Although the kidneys are considered dose-limiting organs [82], Bergsma et al. [121] have shown that renal toxicity remains very low, with no observed grade 3 or 4 toxicity among the 323 patients, and no annual decrease in renal function exceeding 20% for $^{177}{\hbox {Lu}}$-DOTATATE patients. A correlation has been established between the total renal absorbed dose and post-treatment glomerular filtration rate (GFR) [122], which was not demonstrated in the study conducted by Del Prete et al. [123]. Steinhelfer et al. [124] monitored GFR changes in the year following $^{177}{\hbox {Lu}}$-PSMA treatment and demonstrated a decrease of $\ge$ 30% in 23 out of 106 patients.

Regarding the BM, several correlations have been identified in the context of $^{177}{\hbox {Lu}}$-DOTATATE therapy. Decreases in hemoglobin (Hb), platelets (PLT), and white blood cells (WBC) have been found to correlate with the average absorbed dose by the BM per fraction, obtained using a method based solely on planar imaging [104]. Correlations have also been observed between the cumulative absorbed dose to the BM and the decrease in PLT and WBC exclusively [125]. Other studies [90, 123] investigated similar correlations using 3D SPECT acquisitions, considering the vertebrae as substitutes for the BM. However, only the correlation between the variation in PLT and the absorbed dose was identified. The role of the spleen in hematological toxicity for PRRT therapies has also been examined. A correlation between the total absorbed dose by the spleen and the decrease in Hb has been highlighted [105].

Currently, few studies have explored the relationship between the absorbed dose by the BM and hematologic toxicity in $^{177}{\hbox {Lu}}$-PSMA therapy. This may be due to challenges in estimating the absorbed dose by the BM, such as heterogeneity of uptake, dispersed and patient-dependent bone marrow, proximity to highly avid lesions, and destruction of BM during previous treatments [93].

To preserve healthy tissues, thresholds for MTD derived from external beam radiotherapy experience are commonly used for RPT (23 Gy for the kidneys, 2 Gy for the BM), even though the irradiation characteristics differ (dose rates, duration of irradiation, linear energy transfer). These thresholds have been questioned for this type of therapy [121, 125] and may depend on the risk factors presented by the patient [126].

*Dose-response relationship*

For each lesion contouring method presented above, several dose-response correlations have been identified. In $^{177}{\hbox {Lu}}$-DOTATATE therapy, the absorbed dose by individual tumors was found to correlate with tumor reduction for lesions over 2.2 cm in diameter and those over 4.0 cm [127]. Similar correlation was identified by Jahn et al. for pancreatic and small intestinal neuroendocrine neoplasms. Mileva et al. [113] demonstrated improved progression-free survival (PFS) when the lesions received an absorbed dose greater than 35 Gy after the first treatment (C1), along with a reduction of more than 10% in tumor volume expressing SSTR receptors, assessed on the pre-treatment image after C1. For the same radiopharmaceutical, Del Prete et al. [123] found a correlation between the biochemical response (variation in Chromogranin A) and the maximum cumulative absorbed dose by the tumor, but not between the radiological response of the lesion and the cumulative absorbed dose by the lesion.

Unlike $^{177}{\hbox {Lu}}$-DOTATATE therapy, the relationship between absorbed dose in individual lesions and reduction in tumor volume has not been demonstrated for $^{177}{\hbox {Lu}}$-PSMA therapies in the case of low-volume hormone-sensitive metastatic prostate cancers [78]. The correlation was not significant, although the volume of most lymph nodes decreases and that of most bone lesions increases for high absorbed doses. Note that the volumes considered were around 1 mL. Volter et al. [128] showed that absorbed doses by the lesions were significantly higher when a response, as defined by the PERCIST criterion, was observed compared to when there was no response for $^{177}{\hbox {Lu}}$-PSMA-617 therapy. However, the use of this response criterion for PSMA-PET application is controversial, as it was originally developed for FDG-PET [129]. Absorbed doses by individual tumor molecular volumes are correlated with thrombocyte variability, a hemotoxicity biomarker [130] ($^{177}{\hbox {Lu}}$-PSMA I &T). Other authors, such as Violet et al. [21], defined a WB tumor absorbed dose correlated with the PSA response, while Peters et al. [78] correlated it with the absorbed dose by individual lesions. The evolution of PSA levels is affected both by the treatment of certain lesions and the progression of others. Therefore, it is not always possible to establish a correlation between the evolution of PSA levels and response to treatment, as shown by the case study proposed by Murthy et al. [131]. Biological parameters do not always describe the heterogeneity of the disease. Finally, Sgouros et al. [110] provided a review of the technical and biological factors that impact the dose-response relationship in RPT, as well as unresolved issues. A second review proposed by Heidegger et al. [132] focuses on imaging-based and molecular biomarkers to predict treatment response.

## Discussion

This literature review aimed to propose strategies to reduce the burden of imaging in nuclear medicine departments and facilitate the implementation of dosimetry for all patients undergoing RPT with $^{177}{\hbox {Lu}}$. A description of the different steps of the process was provided, followed by proposed interventions to streamline dosimetry procedures: reducing acquisition durations and resources, selecting the type of acquisition, and optimizing acquisition schedules. Certain aspects related to biodistribution and tissue segmentation were described to establish the relationship between absorbed dose, potential toxicities, and treatment response. Tables were also provided, consolidating pusblished dosimetric workflows for $^{177}{\hbox {Lu}}$-PSMA therapies, along with examples for $^{177}{\hbox {Lu}}$-DOTATATE therapies.

### Dosimetry in clinical routine

Post-injection acquisitions are preferably performed during the patient’s hospital stay, which varies depending on the country. For example, in France, patients are discharged after 24 h of hospitalization [54], while in Germany, it is after approximately 2–3 days [34]. The choice of acquisition times results from a compromise between dosimetric precision and clinical constraints (availability of cameras, patient’s return to the hospital), and also depends on the radiopharmaceutical. When the number of acquisitions is reduced to two, studies suggest performing one at 24 h and one at 168 h for $^{177}{\hbox {Lu}}$-DOTATATE therapy, and one at 48 h and one at 168 h for $^{177}{\hbox {Lu}}$-PSMA therapy. If only one acquisition is possible, it should be at least two effective half-lives for the kidneys [32], which is $\sim$96 h [53, 62] for $^{177}{\hbox {Lu}}$-DOTATATE, whereas for $^{177}{\hbox {Lu}}$-PSMA, it should be at $\sim$24–48 h for kidney dosimetry and at $\sim$72–168 h for lesion dosimetry [22, 58]. Generally, a late acquisition (>72 h) is preferred [47], especially when there is only one acquisition, which requires the patient to return to the hospital in any case.

The STP methods presented almost all require information related to a patient population. However, when dosimetry is performed based on a single acquisition, it is not possible to determine if the patient’s pharmacokinetics are similar to that of the population. Estimation errors can be significant and may impact toxicity prediction and therapy personalization. Therefore, it could be valuable to develop criteria to identify patients for whom errors could be significant, in order to propose additional acquisitions. For example, some patients with poor renal function are likely to have slower clearance hence a longer kidney effective half-life that is quite different from the population value. For such cases, potential identified by low eGFR values, dose estimation based on STP imaging should not be recommended.

Implementing voxel-scale dosimetry may be necessary when considering tissue heterogeneity, such as in the case of bone marrow. However, in this scenario, the pharmacokinetics used for each voxel will be assumed to be the same as that of the total tissue. Consequently, between two acquisitions at different time points, the region of the patient contained within a voxel will vary, thus preventing the use of voxel-specific a priori information.

SPECT acquisitions are recommended to improve quantification accuracy, but they can be time-consuming, especially when a large field of view or whole-body imaging is required. Hence, planar acquisitions are often used to reduce scan time (15–20 min vs >1 h for whole-body SPECT acquisition). This is why CZT 360$\circ$ gamma cameras could be a potential solution for obtaining WB SPECT acquisitions with reduced acquisition times due to higher sensitivity (semiconductor technology and innovative camera geometry).

### Patients’ data

Hospitals typically have a limited number of patients with an adequate series of acquisitions in their cohorts, making it challenging to validate simplified methods and assess uncertainties. Therefore, databases that compile data from different centers with varying numbers and acquisition times, as well as different radiopharmaceticals, could facilitate the development of studies with large cohorts and greater data variability (multicenter data). These studies would compare simplified methods to a shared reference protocol, evaluate their performance across various volumes of interest (VOIs), test their applicability with different radiopharmaceuticals, and identify the optimal TP from a broader range of available TPs. The database could also enhance methods that rely on a priori information based on a patient population. Indeed, sub-cohorts of patients could be defined according to their characteristics, and pharmacokinetic parameters could be extracted for each sub-cohort. This approach allows considering patients with significantly different pharmacokinetic parameters than those obtained for the overall population, thereby minimizing uncertainties. Finally, a large patient cohort would be valuable to establish a dose-toxicity relationship, as the toxicities remain low for these patients when the injected activity is 7.4 GBq.

### Maximum tolerated absorbed dose (MTD)

In most studies, the MTD thresholds used have been defined for other therapies and may not apply to RPT. Typically, the calculated absorbed doses are physical doses, expressed in Gy, that do not account for radiobiology, despite its role in toxicities. Determining specific thresholds for each therapy, taking into account the patient’s characteristics and risk factors, therefore seems necessary in order to personalize therapy by limiting toxicities and delivering sufficient dose to the tumors to be treated. Additionally, it would be valuable to investigate whether the implementation of STP methods will have an impact on establishing dose-toxicity relationships.

### Image reconstruction quality

The quality of image reconstruction has a potentially significant impact on absorbed dose estimation. For instance, the partial volume effect spreads counts around the targeted region. However, its correction still needs further study to develop methods that can be easily applied in a clinical setting. Recovery coefficients derived from phantom measurements with spherical objects of varying volume are widely used for PVC, although structures/tumors in the body are not spherical and factors other than volume also impact PVEs. This aspect is more extensively described in the article by Gustafsson et Taprogge [133].

## Conclusions

Many studies have focused on simplified dosimetry methods in $^{177}{\hbox {Lu}}$ therapies to reduce the time and resources allocated to dosimetry, making it easier to implement for all patients. If only two acquisitions are feasible, they should be at 24 h and 168 h for $^{177}{\hbox {Lu}}$-DOTATATE therapy and at 24–48 h and 168 h for $^{177}{\hbox {Lu}}$-PSMA therapy. If only one is possible, it should be around 72–96 h for $^{177}{\hbox {Lu}}$-DOTATATE and 24–48 h for kidneys or 168 h for tumors for $^{177}{\hbox {Lu}}$-PSMA. SPECT are preferred to planar acquisitions for accurate quantification, and the acquisition time can be reduced with 360$^{\circ }$ CZT gamma cameras. Several challenges still need to be addressed: validating and comparing different simplified methods on a large patient cohort and with various radiopharmaceuticals, identifying patients for whom these methods may not be applicable, assessing the impact of STP methods on the development of dose-response relationships, and establishing maximum tolerated dose thresholds for healthy tissues.

## Appendix

See Tables 3 and 4.

**Table 3:** Dosimetric workflows available in the literature for $^{177}{\hbox {Lu}}$-DOTATATE therapies

Authors	N$^\circ$ Patients	Imaging	Time-points	Curve fitting	Dosimetry	VOI	Absorbed dose	Measured T$_{eff}$
							per IA (Gy/GBq)	
Pirozzi Palmese et al.	30	SPECT/CT	3h, 20h	Mono-exp or	MIRD	Kidney	0.54 ± 0.15	NA
(2023) [44]		Discovery	and 90/120h	trapezoidal	formalism	Spleen	0.64 ± 0.32	
		NM/CT		(OAR and T)	OLINDA/	Liver	0.67 ± 0.81	
		670 (GE)		Bi-exp (RM)	EXM$^{\circledR }$v1.0	Red Marrow	0.016 ± 0.006	
						Tumors	4.5 ± 2.9	
Sundlöv et al.	51	Hybrid	WB: 1 h, 24 h,	Trapezoidal	LundADose	Kidneys	0.61 (Median)	51.6 h (median)
(2017) [135]		NA	48 h or 96 h	1 h to 24 h +	software			
			and 168h +	Mono-exp				
			1 SPECT 24h	from 24h				
Del Prete et al.	52	SPECT/CT	4 h, 24 h	Constant (0–4 h)	OLINDA/	Kidney	0.56	NA
(2019) [6]		Symbia T6	and 72h	Linear (4–24 h)	EXM 1.0	Bone marrow	0.031	
				Mono-exp		Tumor	4.8	
Del Prete et al.	36	SPECT/CT	4h, 24h	Trapezoidal	OLINDA	Kidney	0.55 ± 0.19	NA
(2017) [123]		Symbia T6	and 72h	(0–24 h)	software	Bone marrow	0.045 ± 0.025	
		(Siemens)		+ Mono-exp		Tumor	4.0 ± 2.2	
Heikkonen et al.	24	SPECT/CT	24h, 72h	Mono-exp	OLINDA	Whole kidney	0.44 ± 0.15	45.3 ± 5.9h
(2016) [19]		Symbia T2	and 168 h		software	Small volume	0.74 ± 0.28	46.2 ± 5.6h
Sandström et al.	24	*SPECT*	1 h, 24 h,	Mono-exp	OLINDA	Right Kidney	5.5 ± 2.1	NA
(2010) [82]		*Large VOI*	96 h and		Software	Left Kidney	5.0 ± 1.6	
		Hawkeye	168h			Liver	4.5 ± 3.6	
		Millennium (GE)				Spleen	5.8 ± 2.9	
		*SPECT*				Right Kidney	5.3 ± 2.3	
		*Small VOI*				Left Kidney	4.4 ± 1.3	
						Liver	3.1 ± 1.8	
						Spleen	5.7 ± 2.7	
		*Planar*	1 h, 24 h,			Right Kidney	6.7 ± 3.5	
			96h and			Left Kidney	7.8 ± 6.8	
			168h			Liver	4.4 ± 3.2	
						Spleen	5.7 ± 3.2	
Guerriero et al.	28	SPECT/CT	2 h, 6 h,	Best fit	OLINDA/	Kidneys	1.0 ± 0.2	50h (median)
(2013) [32]		Symbia T2	20h, 44h	visually	EXM			
		(Siemens	and 67h	selected				
Del Prete et al.	79	SPECT/CT	4 h, 24 h,	Mono-exp	OLINDA/	Kidney	0.54 (median)	46.6 h (median)
(2018) [52]		Symbia T6	and 72 h		EXM	BM (self-dose)	0.031	72.3 h
						BM (cross-dose)	0.0030	66.9 h
						Tumor (max)	3.8	100.9
Wehrmann et al.	69	Planar	3 h, 20 h,	Mono-exp or	OLINDA/	WB	0.05 ± 0.02	56.1 ± 10.7 h
(2007) [136]		SPIRIT DH-V	44 h and	Bi-exp	EXM	Kidneys	0.9 ± 0.3	63.3 ± 17.5 h
		(Mediso)	68 h			Spleen	1.2 ± 0.5	70.2 ± 16.9 h
						Metastases	9.7 ± 12.4	75.5 ± 20.9 h
						Liver metastases	12.4 ± 15.1	79.0 ± 22.5 h
						Lymph node metastases	8.0 ± 8.4	70.1 ± 18.0 h
						Bone metastases	5.4 ± 4.4	76.2 ± 18.7 h

Authors	N$^\circ$ Patients	Imaging	Time-points	Curve fitting	Dosimetry	VOI	Absorbed dose	Measured T$_{eff}$
							per IA (Gy/GBq)	
						Pancreas metastases	3.0 ± 2.9	71.6 ± 16.0 h
						Soft tissue metastases	5.8 ± 4.0	66.6 ± 20.7 h
Sandström et al.	200	Planar and	24h, 96h	Mono-exp or	OLINDA/	Right kidney	4.69	NA
(2013) [134]		SPECT	and 168h	Bi-exp	EXM	Left kidney	4.39	
						Liver	2.80	
						Spleen	5.35	
Garkavij et al.	21	*SPECT/CT*	24 h and/or	Trapezoidal +	Local	Kidneys (M1A)	0.97 ± 0.24	NA
(2010) [137]		Discovery VH	96h	Mono-exp	deposition	Kidneys (M1B)	1.15 ± 0.29	
		*Planar*	0.5 h, 24 h,		MIRD	Kidneys (M2)	0.81 ± 0.21	
			96 h and 168 h		formalism +	Kidneys (M3)	0.90 ± 0.21	
			Cycles 1–2		Mass-scaled			
			24 h and 96 h					
			Other					
Santoro et al.	12	SPECT/CT	4 h, 24 h,	Mono-exp	OLINDA/	Liver	0.54 ± 0.58	NA
(2018) [138]		Discovery	72h and/		EXM v1.0	Kidneys	0.43 ± 0.13	
		670 NM/CT	or 192h			Spleen	0.61 ± 0.13	
		GE				Red marrow	0.04 ± 0.02	
Marin et al.	47	SPECT/CT	4 h, 24 h	Bi-exp	OLINDA/	Kidneys	0.78 ± 0.35	LK: 55 ± 9h
(2018) [139]		Symbia TruePoint	and 168h		EXM v1.0	Spleen	1.07 ± 0.58	71 ± 9h
		(Siemens)				Red marrow	0.028 ± 0.010	52 ± 18h
Santoro et al.	21	SPECT/CT	4 h, 24 h,	Mono-exp	PLANET Dose	Liver	0.45 ± 0.50	NA
(2021) [38]		Discovery 670	72h and			Kidneys	0.45 ± 0.13	
		NM/CT	192 h			Spleen	0.62 ± 0.17	

For Heikkonen et al. [19], two methods of calculating the absorbed dose to the kidneys are available: whole kidney contouring (whole kidney) and a 4 cm$^{3}$ sphere in the kidney (small volume). For Sandström et al. [82, 134], absorbed doses in Gy are given. NA: Not available

**Table 4:** Dosimetric workflows available in the literature for $$^{177}{\hbox {Lu}}$$-PSMA therapies ($$^{*}$$ $$^{177}{\hbox {Lu}}$$-DKFZ-PSMA-617; $$^{**}$$ $$^{177}{\hbox {Lu}}$$-PSMA-617; $$^{***}$$ $$^{177}{\hbox {Lu}}$$-PSMA-I &T)

Authors	N$^\circ$ Patients	Imaging	Time-points	Curve fitting	Dosimetry	VOI	Absorbed dose	Measured T$_{eff}$
							per IA (Gy/GBq)	
Delker et al.	5	Hybrid	1h, 24h, 48h	Linear and	S-value +	Kidneys	0.6 ± 0.18	NA
(2016) [107]$^{*}$		Symbia T2	and 72h	mono-exp for	organ mass	Salivary glands	1.4 ± 0.53	
		(Siemens)		K, L, S and	SG from planar	Liver	0.11 ± 0.06	
				selected T	images	Spleen	0.10 ± 0.03	
						Bone marrow	0.012 ± 0.005	
						Bone lesions	5.3 ± 3.7	
						Lymph metastases	4.2 ± 5.3	
						ST metastases	2.1 ± 0.8	
Fendler et al.	15	Hybrid	1h, 24h,	Linear and	S-value +	Salivary glands	1.0 ± 0.6	NA
(2017) [142]$$^{**}$$		SPECT patients’	48h and 72h	mono-exp for	organ mass	Right kidney	0.6 ± 0.2	
		head		K, L, S and		Left kidney	0.5 ± 0.3	
				selected T		Tumor	6.1 ± 4.9	
						Liver	0.1 ± 0.1	
						Spleen	0.1 ± 0.1	
						Bone marrow	0.002 ± 0.005	
Yadav et al.	26	Planar	0.5h, 3.5h, 24h,	Mono-exp or	OLINDA/EXM	Liver	0.36 ± 0.11	NA
(2017) [143]$^{*}$		Symbia	48h, 72h, 96h,	Bi-exp	1.0	Kidneys	0.99 ± 0.31	
		(Siemens)	120h, 144h			Salivary glands	1.24 ± 0.27	
			and 168h			Red marrow	0.048 ± 0.059	
						Bladder + contents	0.23 ± 0.092	
						Tumors	10.94 ± 18.01	
						WB	0.016 ± 0.003	
Violet et al.	30	2-3 SPECT/CT	4 h, 24 h	Tri-exp	OLINDA	Parotid glands	0.58 ± 0.43	NA
(2019) [21]$$^{**}$$		Symbia T6 or	and 96h			Submandibular glands	0.44 ± 0.36	
		Intevo 16				Lacrimal glands	0.36 ± 0.18	

Authors	N$^\circ$ Patients	Imaging	Time-points	Curve fitting	Dosimetry	VOI	Absorbed dose	Measured T$_{eff}$
							per IA (Gy/GBq)	
		(Siemens)				Kidneys	0.39 ± 0.15	
						Spleen	0.08 ± 0.06	
						Liver	0.10 ± 0.05	
						Bone marrow	0.11 ± 0.10	
						Bone metastases	5.28 ± 2.46	
						Node	3.91 ± 3.93	
Kabasakal et al.	7	Planar	4h, 24h,	Bi-exp	OLINDA 1.1	Parotid glands	1.17 ± 0.31	
(2015) [144]$$^{**}$$		Symbia T16	48 h and			Kidneys	0.88 ± 0.40	
		(Siemens)	120 h			Bone marrow	0.034 ± 0.014	
						Liver	0.28 ± 0.09	
						WB	0.061 ± 0.026	37.9 ± 14.6h
Okamoto et al.	18	Planar	30-120 min,	Mono-exp or	OLINDA/	Kidneys	0.72 ± 0.21	NA
(2017) [108]$$^{***}$$		Symbia T6	24h and	Bi-exp	EXM	Liver	0.12 ± 0.06	
		(Siemens)	6-8d			Parotid glands	0.55 ± 0.14	
						Lacrimal glands	3.8 ± 1.4	
						Submandibular glands	0.64 ± 0.40	
						Tumors	3.2 ± 2.6	
						Lymph node metastases	3.2 ± 2.2	
						Bone metastases	3.4 ± 2.7	
						Liver metastases	1.2 ± 0.67	
						Lung metastases	1.75 ± 0.92	
Feuerecker et al.	6	Planar	1h, 4h,	Mono-exp or	OLINDA/	Bone marrow (L2-L4)	0.22 ± 0.21	NA
(2022) [109]$$^{***}$$		Symbia T6	24h, 48h	Bi-exp	EXM	Bone marrow (thigh)	0.30 ± 0.27	
		(Siemens)	and 7d			Kidneys	0.73 ± 0.18	
						Liver	0.07 ± 0.03	
						Parotid glands	0.80 ± 0.41	
						Lacrimal glands	1.92 ± 0.80	
						Submandibular glands	0.67 ± 0.31	

Authors	N$^\circ$ Patients	Imaging	Time-points	Curve fitting	Dosimetry	VOI	Absorbed dose	Measured T$_{eff}$
							per IA (Gy/GBq)	
						Tumor	2.64 ± 2.24	
						Bone metastases	1.70 ± 1.13	
						Lymph node metastases	4.51 ± 2.69	
Scarpa et al.	10	Hybrid	0.5h, 4h,	Mean of two	OLINDA/	Red marrow	0.042 ± 0.028	NA
(2017) [145]$$^{**}$$		Symbia	24 h, 72 h	or three exp	EXM	Lacrimal glands	1.0 ± 0.69	
		(Siemens)	and 96h	decay functions		Parotid glands	0.56 ± 0.25	
			+24 h (SPECT)			Submandibular glands	0.50 ± 0.15	
						Kidneys	0.60 ± 0.36	
						Spleen	0.12 ± 0.09	
						Liver	0.12 ± 0.06	
						Skeletal metastases	3.4 ± 1.9	
						Lymph node metastases	2.6 ± 0.4	
						Visceral metastases	2.4 ± 0.78	
Peters et al.	10	SPECT/CT	1h, 24h,	Trapezoidal +	Hermes	Salivary glands	0.39 ± 0.17	32.5 h (median)
(2022) [78]$$^{**}$$		Symbia T16	48h, 72h	Mono-exp or	HybridViever	Kidneys	0.49 ± 0.11	28.4h
		(Siemens)	and 168 h	Bi-exp or		Lesions	3.25 ± 3.19	62 h
				Tri-exp +		Liver	0.09 ± 0.01	19.0 h
				extrapolation		Bone marrow	0.017 ± 0.008	12.5h (blood)$^{1}$
Zhang et al.	16	Hybrid	5 WB between	Bi-exp	OLINDA/	Kidneys	0.81 ± 0.32	NA
(2019) [146]$$^{**}$$		SPIRIT DH-V	0.5–118 h + 1 SPECT		EXM	WB	0.058 ± 0.027	
		(Mediso)	between 45–118 h			Tumor	13.75 ± 31.59	
Kabasakal et al.	6	Hybrid	WB: 4 h, 24 h,	NA for OAR	OLINDA	Parotid glands	1.90 ± 1.19	NA
(2017) [147]$$^{**}$$		Symbia T16	48 h and 120 h	Bi-exp for	1.1	Kidneys	0.82 ± 0.25	
		(Siemens)	+ 1 SPECT 24 h	blood		Bone marrow	0.030 ± 0.008	

Authors	N$^\circ$ Patients	Imaging	Time-points	Curve fitting	Dosimetry	VOI	Absorbed dose	Measured T$_{eff}$
							per IA (Gy/GBq)	
						Liver	0.17 ± 0.09	
Sarnelli et al.	9	Planar	1 h, 16–24 h,	Mono-exp or	OLINDA/	Parotid glands	0.81 ± 0.74	33.0 h (median)
(2019) [148]$$^{**}$$		Discovery NM/CT	36–48 h and	Bi-exp	EXM 1.1	Kidneys	0.67 ± 0.27	31.4 h
		670 (GE)	120h			Red marrow	0.044 ± 0.017	8.2h
						WB	0.049 ± 0.031	40.1 h
						Liver	0.16 ± 0.15	25.4 h
Rosar et al.	24	SPECT/CT	24 h, 48 h	Constant +	IDAC 2.1	Kidneys	0.54 ± 0.28	NA
(2021) [20]$$^{**}$$		Philips BrightView	and $$\ge$$ 96h	Trapezoidal +	software	Liver	0.10 ± 0.05	
		XCT (Philips)		Trapezoidal +		Parotid glands	0.81 ± 0.34	
				Mono-exp		Submandibular glands	0.72 ± 0.39	
						Bone metastases	1.68 ± 1.32	
Paganelli et al.	13	Hybrid	WB: 0.5–1 h, 16–24 h,	Mono-exp (L),	OLINDA/	Parotid glands	1.04 ± 0.82	NA
(2020) [96]$$^{**}$$		Discovery NM/CT	36-48h and	Bi-exp (PG, K, RM,	EXM 1.0	Kidneys	0.41 ± 0.19	
		670 (GE)	120h + 1 SPECT/CT	WB, T) and		Liver	0.18 ± 0.14	
			16-24h	Tri-exp (T)		Submandibular glands	0.67 ± 0.36	
						Lacrimal glands	2.06 ± 1.24	
						Red marrow	0.042 ± 0.017	
						WB	0.053 ± 0.037	
						Bone lesions	4.70 (mean only)	
						Lymph node lesions	3.64 (mean only)	
Kratochwil et al.	4	Hybrid	WB: 0.5h, 3h,	Three phases:	OLINDA/	Kidneys	0.75 ± 0.19	NA
(2016) [140]$$^{**}$$		GE Hawkeye	20h, 44h and	linear + mono- or	EXM	Red marrow	0.03 ± 0.01	
		Millennium	5–8 d SPECT: 20h	bi-exp + mono-		Parotid glands	1.28 ± 0.40	
				or Bi-exp		Submandibular glands	1.48 ± 0.37	

Authors	N$^\circ$ Patients	Imaging	Time-points	Curve fitting	Dosimetry	VOI	Absorbed dose	Measured T$_{eff}$
							per IA (Gy/GBq)	
						Metastases	6–22	
Baum et al.	30	Hybrid	5 WB between	Mono-exp or	OLINDA/	WB	0.02 ± 0.01	37 ± 19 h (median)
(2016) [149]$^{\dagger }$		SPIRIT DH-V	0.5h-118h + 1 SPECT	Bi-exp	EXM	Kidneys	0.8 ± 0.4	33 ± 14h
		(Mediso)	between 45h-118h			Parotid glands	1.3 ± 2.3	25 ± 5h
						Tumors	3.3 ± 14	51 ± 30h
						Bone metastases	3.0 ± 10	52 ± 30h
						Lymph node metastases	4.0 ± 20	43 ± 32h
Hohberg et al.	9	Planar	0.5h, 24h,	Bi-exp	OLINDA/	Lacrimal glands	2.82 ± 0.76	NA
(2016) [101]$^{*}$		ECAM	48h, 72h		EXM 1.1	Salivary glands	0.72 ± 0.14	
		(Siemens)	and 168h			Kidneys	0.53 ± 0.17	
						Nasal mucous membrane	0.42 ± 0.12	
						WB	0.063 ± 0.023	
Mix et al.	59	SPECT/CT	1 h, 24 h,	Trapezoidal +	STRATOS	Kidneys	0.67 ± 0.24	NA
(2022) [43]$$^{**}$$		BrightView XCT	48 h, 72 h	exponential tail				
			and 6-9d					
Prive et al.	10	SPECT/CT	1 h, 24 h,	NA	MIRD	Salivary glands	0.39 ± 0.17	NA
(2021) [150]$$^{**}$$		Symbia T16 or	48 h, 72 h			Kidneys	0.49 ± 0.11	
		Intevo Bold	and 168h			Liver	0.09 ± 0.01	
						Bone marrow	0.02 ± 0.00	
						Tumor (Cycle 1)	2.51 ± 2.43	
						Tumor (Cycle 2)	1.78 ± 1.24	
Völter et al.	30	SPECT (CT 24h)	24 h, 48 h,	Mono-exp	Mass-scaled	Tumor	5.7 ± 6.4	NA
(2021) [128]$$^{**}$$		Symbia T2	and 72h		sphere S-value	Lymph node metastases	7.7 ± 9.7	
		(Siemens)				Bone metastases	4.7 ± 3.9	
Barna et al.	22	Planar	0.5 h, 4 h,	Linear + Mono-exp	IDAC-Dose 2.1	Kidneys	0.71	NA
(2020) [130]$$^{***}$$		GE Discovery VH	18h, 24h,	(PG) or Bi-exp		Liver	0.27	
		GE Millennium VG	48h, 72h	(Other)		Red marrow	0.040	
		Siemens E.CAM	and/or 96h			Tumors (bone)	4.4	

Authors	N$^\circ$ Patients	Imaging	Time-points	Curve fitting	Dosimetry	VOI	Absorbed dose	Measured T$_{eff}$
							per IA (Gy/GBq)	
						Tumor (Lymph node)	5.5	
						Tumor (Liver)	4.9	
						Parotid glands	0.77	
Maffey-Steffan et al.	32	Planar	0.5h, 4h,	Tri-exp decay	OLINDA/	Bone metastases	4.3 ± 3.0	NA
(2020) [151]$$^{**}$$		Symbia	24h, 72h	or Bi-exp for	EXM	Lymph node metastases	3.3 ± 2.2	
		(Siemens)	and 96h	WB-remainder		Visceral lesions	3.0 ± 1.4	
						Red marrow	0.039 ± 0.028	
						Lacrimal glands	0.85 ± 0.51	
						Parotid glands	0.53 ± 0.22	
						Submandibular glands	0.46 ± 0.17	
						Kidneys	0.77 ± 0.56	
						Liver	0.13 ± 0.08	
Schuchardt et al.	138	Planar	5 WB between	Mono-exp or	OLINDA	Kidneys	0.77$$^{**}$$/0.92$$^{***}$$	40h$$^{**}$$/33h$$^{***}$$
(2022) [141]		SPIRIT DH-V	0.5h to 68h	Bi-exp	2.0	WB	0.04/0.03	42h$$^{**}$$/35h$$^{***}$$
		(Mediso)	1 SPECT at			Parotid glands	0.5/0.5	31h$$^{**}$$/23h$$^{***}$$
		Symbia T	24 h, 48 h			Lacrimal glands	5.1/3.7	28h$$^{**}$$/25h$$^{***}$$
		(Siemens)	or 72 h			Tumor	5.9/5.8	61 h$$^{**}$$/43 h$$^{***}$$
						Bone metastases	6.0/5.9	60h$$^{**}$$/43h$$^{**}$$
						Lymph node metastases	7.1/6.9	55 h$$^{**}$$/42h$$^{***}$$
Ozkan et al.	10	Planar	4h, 24h,	Exponential	OLINDA/	Kidneys	0.70 ± 0.24	NA
(2020) [152]$$^{***}$$		JETStream	48 h, 72 h,	curves	EXM 1.1	Parotid glands	1.34 ± 0.78	
		(Philips) or	120h and 168h			Submandibular glands	0.94 ± 0.45	
		Intevo 6				Lacrimal glands	2.28 ± 1.29	

Authors	N$^\circ$ Patients	Imaging	Time-points	Curve fitting	Dosimetry	VOI	Absorbed dose	Measured T$_{eff}$
							per IA (Gy/GBq)	
		(Siemens)						
Kamaldeep et al.	30	Planar	0.5h, 2h,	Bi-exp	OLINDA 2.0	Kidneys	0.49 ± 0.17	NA
(2021) [153]$$^{**}$$		Symbia E	12h, 24h,			Liver	0.07 ± 0.04	
		(Siemens)	72/96h			Salivary glands	0.53 ± 0.25	
						Lacrimal glands	1.23 ± 0.70	
						Bone marrow	0.03 ± 0.02	
						Spleen	0.16 ± 0.08	
						Bone lesions	6.03 ± 8.34	
						Lymph nodes	15.71 ± 14.72	
						Primary site	3.29 ± 2.76	
						Liver lesions	9.92 ± 3.02	
						Lung lesions	5.30 ± 8.22	
						Soft tissue deposit	4.68 ± 4.81	
Xue et al.	23	Hybrid	WB: 30-150 min	NA	OLINDA/	WB	0.031 ± 0.017	NA
(2022) [154]$$^{***}$$			24h and 6-8d		EXM	Kidneys	0.648 ± 0.165	
						Liver	0.067 ± 0.035	
						Salivary glands	0.565 ± 0.389	
						Spleen	0.306 ± 0.227	
Chatachot et al.	8	Planar	4h and 24h	Mono-exp	OLINDA/	Kidneys	0.81 ± 0.24	NA
(2021) [155]$$^{***}$$		Discovery			EXM v. 2.0	Bone marrow	0.02 ± 0.01	
		NM/CT 670				Liver	0.13 ± 0.10	
		(GE)				Urinary bladder	0.27 ± 0.25	
						Spleen	0.16 ± 0.07	
						Lacrimal glands	3.62 ± 1.78	
						Parotid glands	0.21 ± 0.14	
						Submandibular glands	0.09 ± 0.07	
Peters et al.	10	SPECT/CT +	1 h, 24 h, 48 h,	Mono-exp		Kidneys		39 h ± 5 h
(2023) [58]		Planar (Symbia T16	72 h, and 168 h			Liver		29 h ± 8 h
		or Intevo Bold				Salivary glands		33 h ± 4 h
						Tumors		6 9h ± 13 h

$^{1}$Measured T$_{eff}$ of the main component. For Kratochwil et al. [140], a trapezoidal model was used to fit the salivary glands and tumors. $^{\dagger }$ median±std For Schuchardt et al. [141], mean doses are given for $^{177}{\hbox {Lu}}$-PSMA-617 (left) and $^{177}{\hbox {Lu}}$-PSMA-I &T (right). ST: Soft Tissue NA: Not Available

## Footnotes

1 https://pubmed.ncbi.nlm.nih.gov/.

2 https://clinicaltrials.gov/.
